# Radical Resection for Locally Advanced Colon Cancer With Bladder Involvement Treated in a Tertiary Health Care Centre

**DOI:** 10.7759/cureus.54333

**Published:** 2024-02-16

**Authors:** Anilreddy Cherukula, Suraj Agrawal, Suhas N Jajoo, Garima Saxena

**Affiliations:** 1 Department of General Surgery, Datta Meghe Institute of Medical Sciences, Wardha, IND; 2 Department of Surgery, Jawaharlal Nehru Medical College, Acharya Vinoba Bhave Rural Hospital, Wardha, IND; 3 Department of General Surgery, Jawaharlal Nehru Medical College, Datta Meghe Institute of Medical Sciences, Wardha, IND

**Keywords:** adenocarcinoma of the sigmoid colon, chemotherapy, partial pystectomy, high anterior resection, urinary bladder invasion

## Abstract

Colorectal cancer with involvement of the urinary bladder is infrequent in the nonmetastatic setting. Procedures for advanced colorectal cancers with bladder involvement may include partial or complete bladder resections. Proper therapeutic management principles dictate radical surgery when negative margins can be obtained. High-resolution CT imaging along with endoscopic evaluation of the urinary bladder is frequently required to assess the extent of urinary bladder dissection. Here, we present a case of adenocarcinoma of the sigmoid colon with urinary bladder involvement and its treatment.

## Introduction

Upon presentation, nearly 5-22% of all colorectal cancers are locally advanced. These subtypes of colorectal cancer are distinguished by aggressive local behaviour, such as invasion of nearby organs or structures, and the absence of distant metastasis at the time of presentation. These patient groups have comparable survival rates to traditional resections [1]. In 3-10% of cases, the genitourinary tract (vagina, urinary bladder, ureters and prostate) is infiltrated [2-4]. Colorectal cancer most commonly affects the urinary bladder, followed by the rectum. Total pelvic exenteration (TPE) with urinary diversion or wide local excision with bladder-sparing procedures are available in cases where the bladder is involved [5-6]. The importance of obtaining negative surgical microscopic margins in preventing local recurrences was a recurring finding [7,8]. The lymph node status was the single best predictor of overall survival in patients undergoing aggressive surgical resection, followed by the specimen margin status [8-10]. We discuss our approach to treating operable locally advanced colorectal bladder carcinoma.

## Case presentation

**Figure 1:**
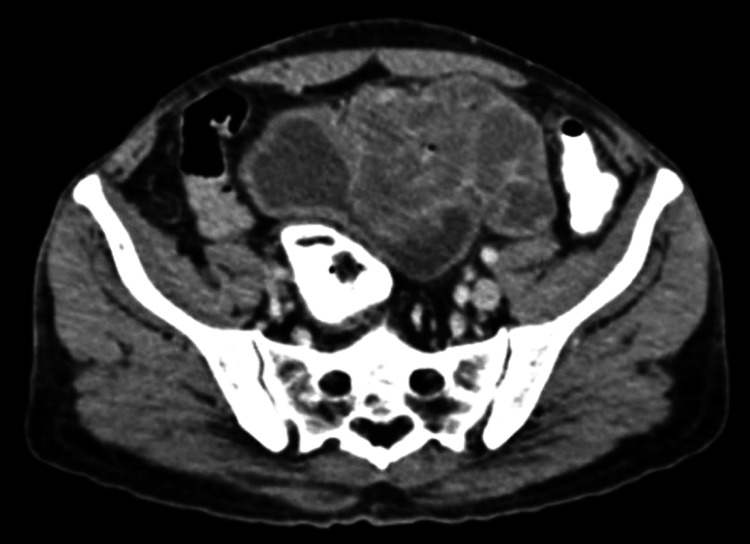
Contrast-enhanced computed tomography (CECT) image showing the sigmoid colon mass extending into the urinary bladder

A 56-year-old man from rural Maharashtra, India, presented to our hospital with complaints of a lump in the lower abdomen associated with multiple episodes of vomiting, weight loss and dark-coloured stools, two to three episodes of vomiting per day in the last five days and weight loss of 5 kg in the last month. He also complained of dark-coloured stools for the last three days. Upon blood investigations, his tumour markers were cancer embryonic antigen (CEA) of 326 ng/dl and carbohydrate antigen (CA) 19-9 of 1.4 U/ml. Contrast-enhanced computed tomography (CECT) imaging revealed a long-segment homogenously enhancing circumferential asymmetric bowel wall thickening with a maximum thickness of 2.5 cm over a length of approximately 10 cm with aneurysmal dilatation of the sigmoid and descending colon. However, the proximal and distal bowel loops appeared normal in calibre. There is a loss of fat planes in the superior border of the urinary bladder with the extension of the lesion into the bladder (depth of invasion = 4 mm)and adjacent thickening of the bladder wall (6.2 mm) with adjacent lymphadenopathy most likely suggestive of neoplastic origin (Figure 1).

**Figure 2:**
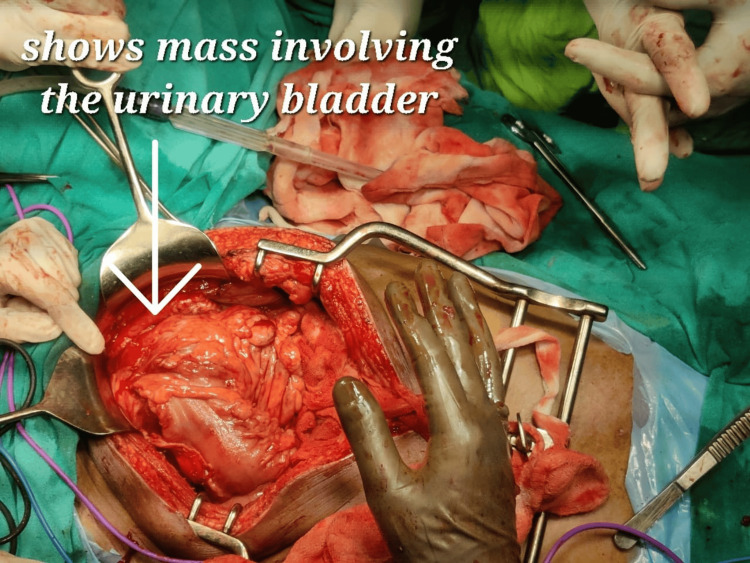
Intraoperative image showing a mass in the sigmoid colon region involving the bladder

Colonoscopy shows large circumferential friable ulcers with a proliferative growth in the sigmoid colon suggestive of sigmoid colon growth. Multiple biopsies were taken from the mass, and the histopathological report revealed an adenocarcinoma of the sigmoid colon. CECT thorax was also done to know the metastatic status, revealing a normal study. A tumour board discussion was done, which advised surgical management. The patient was then taken to the operation theatre, and intraoperatively, a mass of 8 x 6 cm, hard in consistency involving the sigmoid colon and invading dome of the bladder, was observed (Figure 2).

**Figure 3:**
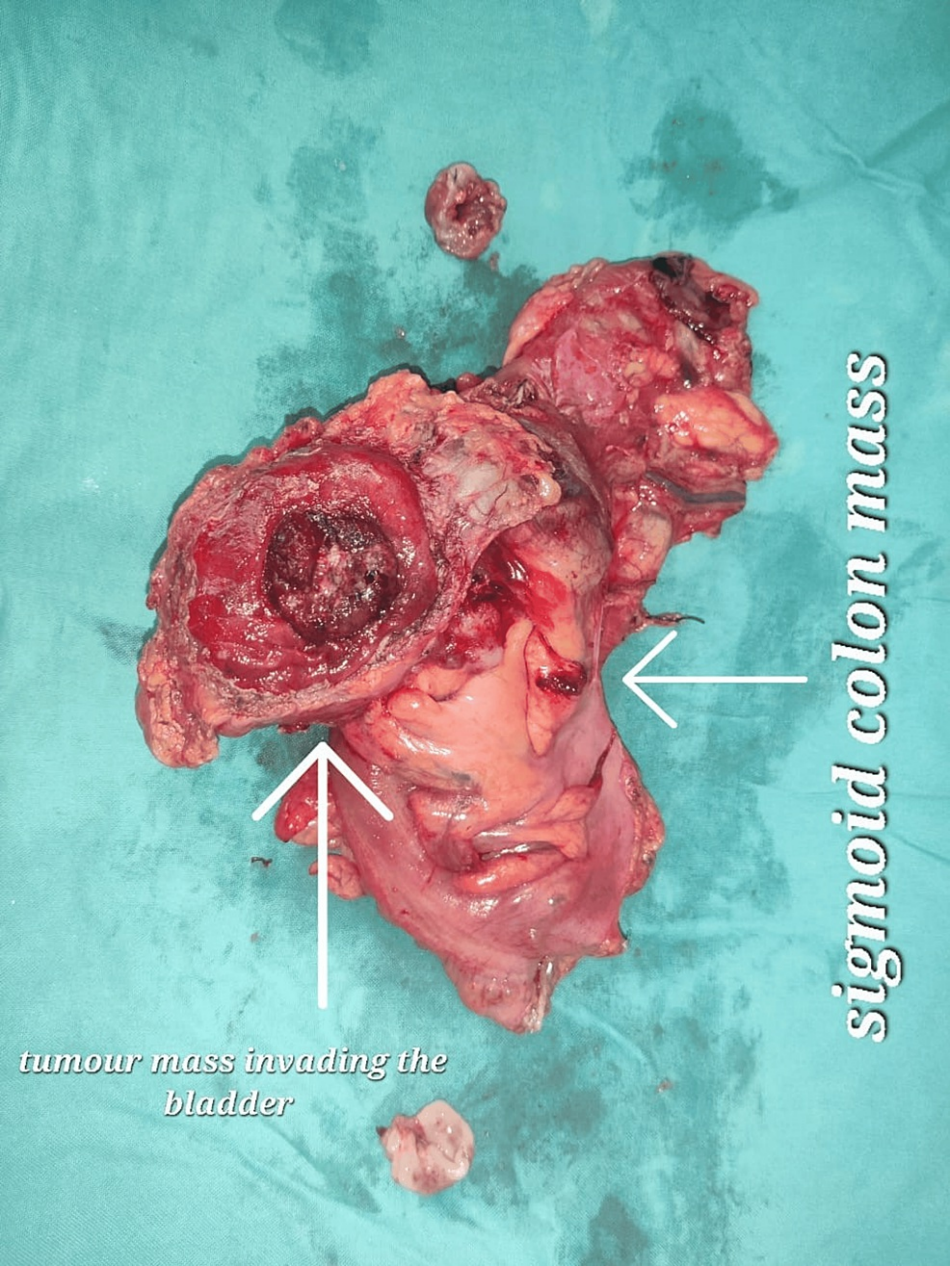
Intraoperative imaging showing a resected specimen of the sigmoid colon mass with the involvement of the dome of the urinary bladder

The colonic mass was free from the surrounding structure like the left ureter and bilateral iliac vessels, so a high anterior resection with an apical clearance was done, and the dome of the urinary bladder was removed en bloc with the tumour, keeping a 2 cm margin around the tumour (Figure 3).

**Figure 4:**
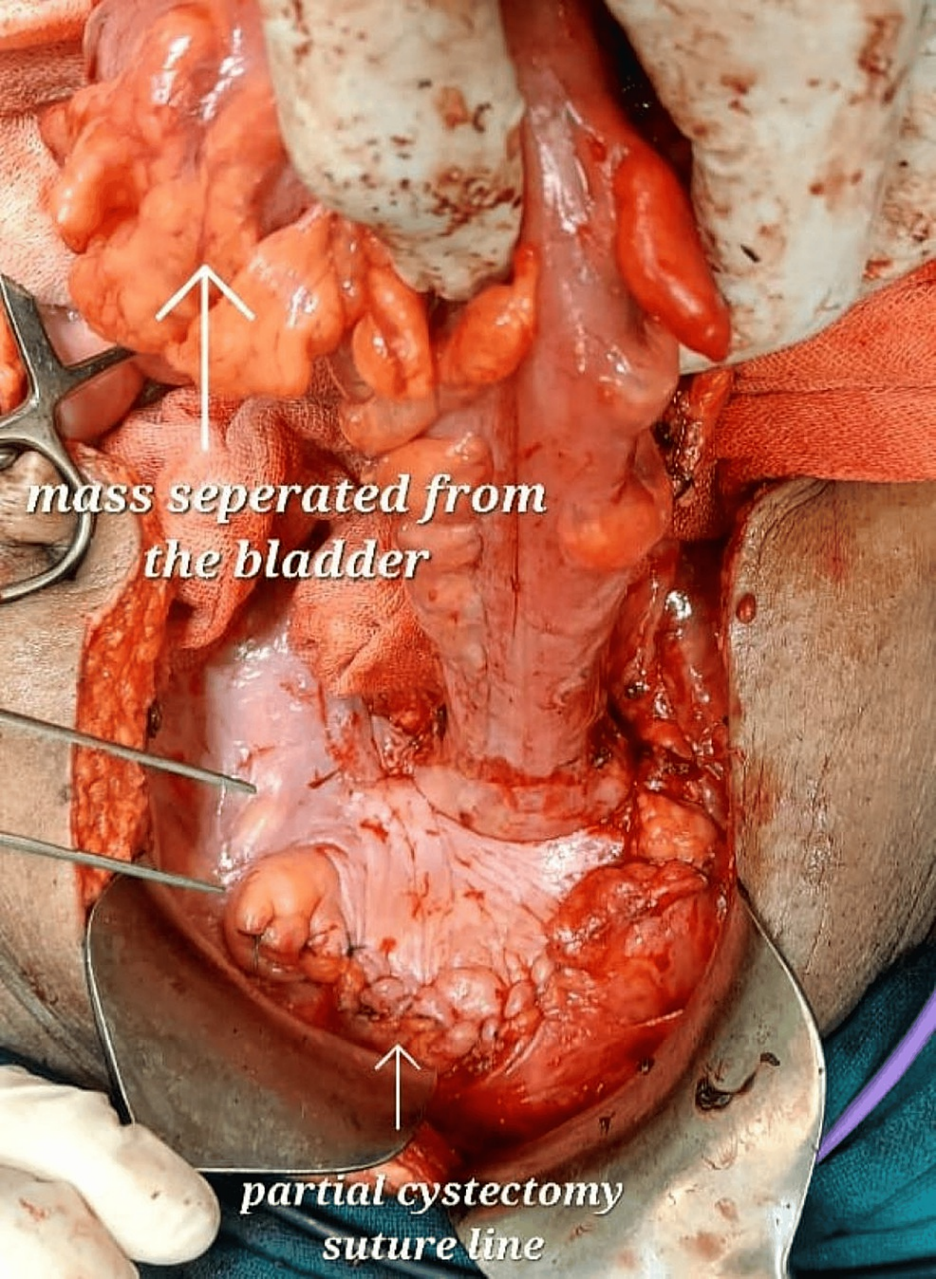
Intraoperative image showing the mass separated from the bladder and suture line after partial cystectomy

The residual bladder was large enough for a satisfactory voiding function; hence, the decision was made to repair the urinary bladder in two layers (Figure 4).

**Figure 5:**
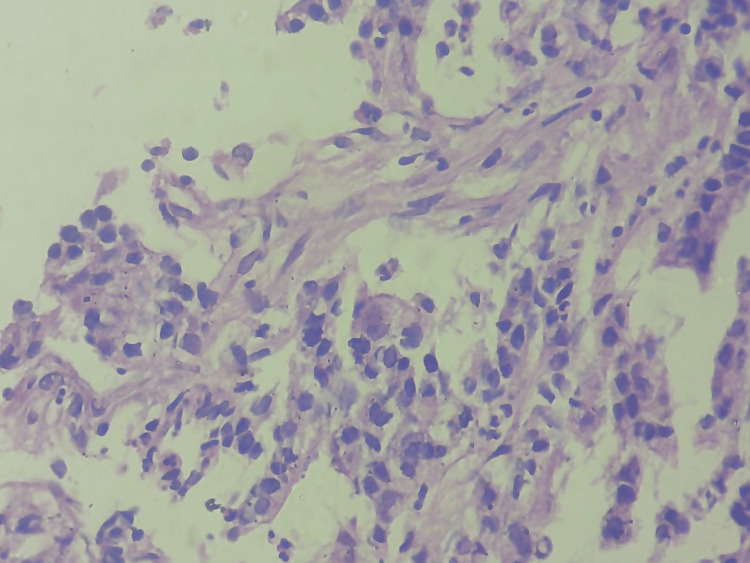
Section stained with haematoxylin and eosin (H&E) stain (high power view: 40x) shows poorly formed glandular structures lined by malignant epithelial cells, which are polygonal, with an abundant cytoplasm and pleomorphic, hyperchromatic nuclei. Histopathological features suggestive of adenocarcinoma.

Bladder wash was given, and bladder closure was done in layers. The end-to-end colorectal anastomosis was done using a circular stapler, and the specimen was sent for histopathological examination. The histopathology report shows a tumour of size 9.5 x 7.5 x 6 cm. The tumour directly invades the dome of the bladder (invades beyond the serosa into the adjacent organs, suggestive of grade II moderately differentiated mucinous adenocarcinoma; Figure 5).

The tumour shows a lymphovascular invasion, and without perineural invasion and high budding score, a total of 32 lymph nodes were identified, among which one is positive. The TNM staging of the tumor is pT4b pN1a pMx (stage III ). The patient had an uneventful recovery and was discharged on postoperative day 10. Foley's catheter was removed after three weeks, and the patient had satisfactory voiding. Later, he was given adjuvant chemotherapy.

## Discussion

The sigmoid colon and rectum are the locations where primary tumours that invade the bladder occur more frequently compared to other colon segments [4,11]. Even in patients of locally advanced colorectal cancer with a colovesical fistula, extensive surgery with en bloc bladder resection increases local control and survival [12]. The prognosis is mostly determined by a negative surgical margin and the existence or absence of a colovesical fistula [13,14]. Extended resection, on the other hand, necessitates partial or total bladder resection with urinary tract diversion. In this case, preserving the bladder was possible because the tumour had invaded the bladder dome over a small area permitting wide margins and sufficient bladder volume. Partial bladder resection should be considered to preserve the bladder. If the tumour involves the bladder trigone area, then radical cystectomy with urinary diversion is required. Following that, a precise radiological diagnosis along with cystoscopic evaluation is advised prior to surgery in order to accurately assess the extent of the surgical resection.

Surgical resection is the main and best therapy for advanced colon cancers [15]. Prior to surgery, a full discussion about urinary repair or diversion should take place. All these patients should be discussed in the multidisciplinary tumour board to decide the optimal line of treatment. Bladder-sparing approaches have been shown to produce equivalent oncologic results with much less morbidity [16]. Complete excision of the affected tissues is still necessary to get the desired results.

The bladder can be repaired primarily or augmented with a bowel segment if the residual bladder volume is insufficient (enterocystoplasty). Patients with poor prior compliance and symptomatic bladder overexpression brought on by radiotherapy and those who experienced a considerable decrease in bladder volume following surgery should be given enterocystoplasty consideration. The amount of bladder tissues removed after surgery is an arbitrary and challenging sign to measure. Some individuals will be largely closed (without augmentation) and experience symptomatic storage issues, necessitating subsequent augmentation. This is due to the hazards of augmentation and the potential for secondary augmentation (at a later time). Many scholars advocate starting off cautiously. After partial cystectomy, the bladder's capacity may be decreased to 50-100 mL, and a negative oncologic margin is necessary [17]. Just the bladder neck and trigone may be spared in extreme situations (supratrigonal cystectomy). Enterocystoplasty could be a good substitute for urine diversion in some circumstances. Bladder augmentation can increase compliance and functional status, but it can also cause voiding dysfunction that necessitates self-catheterization. When treating advanced (T3-T4) colorectal cancer, exenteration or complete cystectomy may be necessary with urinary diversion. The technique of urinary diversion must be chosen after taking into account a variety of criteria. When choosing a urinary diversion approach, take into account the patient's aspirations, comorbidities, operational history and the specialist's expertise with various diversion techniques. The optimal urine diversion has not been determined by quality-of-life studies [18]. It is still routine practise to employ ileal or colon conduits for non-continent urine diversion. This is particularly true for challenging cases, such as those who have advanced non-urologic malignancies that need for pelvic exenteration and maybe radiation therapy. More than 75% of patients undergoing radical cystectomy use the ileal conduit at numerous tertiary high-volume referral centers [18].

In patients with a healthy bladder that is functioning properly and a single tumour that is within the range of a 1- to 2-cm resection margin, partial cystectomy is recommended. In some circumstances, locally advanced malignancies of the rectal, colon, prostate, uterine, cervix or ovary can be treated with a partial cystectomy, but pelvic exenteration may be necessary. For at least two years after a partial cystectomy for bladder cancer, patients should receive a cystoscopy and a cytologic urine test every three months. In the first several years of follow-up, routine pelvic and abdominal CT scans are advised. With negative surgical margins, a 17% local recurrence rate and a 39-74% three-year survival rate have been recorded. Although it has been demonstrated that patients with muscle-invasive bladder cancer (MIBC) benefit from platinum-based neoadjuvant chemotherapy (NCT), the ideal neoadjuvant regimen has not yet been determined. Two of the most popular chemotherapy regimens in contemporary oncology are gemcitabine and cisplatin/carboplatin (GC) and methotrexate, vinblastine, doxorubicin, and cisplatin (MVAC)/gemcitabine and cisplatin/carboplatin (GC). An improved and more accurate assessment of the survival benefit of cisplatin-based NCT in MIBC is given in this two-step meta-analysis. This study also showed that, in the neoadjuvant situation, MVAC could have a better overall survival rate than GC (with or without carboplatin data). The results indicate that NCT should be standard treatment for MIBC and that MVAC may be the best neoadjuvant regimen.

## Conclusions

Sigmoid colon cancer with partial involvement of the urinary bladder can be managed with anterior resection and partial cystectomy so that cystectomy and morbidity associated morbidity. With proper surgery and adjuvant care, even advanced cancer can be cured. Moreover, even if there is a recurrence, it can be identified early so that early interventions can be taken.
